# Whole-Genome Sequence of the Endophytic Streptomyces sp. Strain CBMAI 2042, Isolated from Citrus sinensis

**DOI:** 10.1128/MRA.01426-18

**Published:** 2019-01-10

**Authors:** Luciana Gonzaga de Oliveira, Renata Sigrist, Bruno Sachetto Paulo, Markyian Samborskyy

**Affiliations:** a Department of Organic Chemistry, University of Campinas, Campinas, São Paulo, Brazil; b Department of Biochemistry, University of Cambridge, Cambridge, England; University of California, Riverside

## Abstract

The whole-genome sequence of Streptomyces sp. strain CBMAI 2042, an endophytic actinobacterium isolated from Citrus sinensis branches, is described. The strain has the ability to inhibit the growth of Xylella fastidiosa and other human pathogens. *In silico* analysis highlighted the presence of nonribosomal peptide and polyketide synthases, revealing promising antibiotic assembly lines.

## Abstract (precis)

The whole-genome sequence of Streptomyces sp. strain CBMAI 2042, an endophytic actinobacterium isolated from Citrus sinensis branches, is described.

## ANNOUNCEMENT

Streptomyces sp. strain CBMAI 2042 is an endophyte from Citrus sinensis capable of inhibiting the growth of Bacillus megaterium, Staphylococcus aureus, and Candida albicans pathogens (1). It is also known as a Xylella fastidiosa inhibitor, promoting the degradation of xantham gum (2) and other pathogens of Citrus subsp., such as Geotrichum candidum var. citri-aurantii and Colletotrichum gloeosporioides, representing an affordable alternative for sustainable agriculture practice (3). This actinobacterium was isolated from plant branch tissues. After surface sterilization, bark-free fragments (4 to 6 mm long) were plated onto petri dishes containing tryptic soy agar (TSA) supplemented with benomyl (50 µg ml^−1^) for fungal growth inhibition. The plates were incubated at 28°C for 20 days (2). This endophyte was isolated from the cut pieces and identified based on the closest relative 16S rRNA matches (Streptomyces anuatus; the locus_tag prefix is STAN).

For high-molecular-weight genomic DNA (gDNA) extraction, a single colony was transferred to 50 ml of tryptic soy broth containing yeast extract (TSBY; Oxoid) and incubated at 28°C for 48 h. gDNA extraction followed the salting-out procedure (4), yielding nearly 3,000 ng μl^−1^. The whole-genome draft was generated using Illumina shotgun TruSeq PCR-free and Nextera mate pair library prep using the manufacturer’s protocol. Sequencing was carried out using V2 Illumina sequencing chemistry and run on a MiSeq instrument (2 × 250-bp paired-end [PE] sequencing).

The Illumina shotgun library produced 1.5 million reads in a total of 0.6 GB of data. The mate pair library produced 3.7 million reads in a total of 1 GB of data. The shotgun data were preprocessed without Illumina adapter sequences and used for bcl2fastq conversion. Reads were processed using an in-house Illumina adapter-trimming tool (fastq_miseq_trimmer) (5). The paired reads from the adapter-trimmed files were then preassembled using FLASh v1.2.11 (https://ccb.jhu.edu/software/FLASH/). The resulting combined read and not-combined read files were fed into Newbler v3.0 (Roche, 454 Life Sciences) for *de novo* assembly. The mate pair data set was preprocessed following the same workflow (fastq_miseq_trimmer tool), and the resulting split mate pair reads were assembled using Newbler v3.0. The assembly was then subjected to iterative Pilon polishing (v1.13), converted to Consed-compatible ace format, and checked using Consed (6, 7), generating 3 scaffolds, consisting of 53 contigs. This pipeline afforded 99% of the total genome coverage. The total genome size comprises 8,211 kbp (total length, 8,211,326 bp), with a G+C content of 68.4%.

Annotation was carried out using a customized pipeline based on FgeneSB as the open reading frame (ORF) predictor, operating in the *ab initio* mode (M. Samborskyy, unpublished data). The annotation results were edited using Artemis (8). Rapid Annotations using Subsystems Technology (RAST) server annotation (9) allowed for the identification of 7,198 candidate protein-coding genes. Additionally, 17 transcription factor initiators and subsystems related to the biosynthesis of secondary metabolites, fatty acids, lipids, isoprenoids, and siderophores were identified. Analysis through the antiSMASH 3.0.1 (10, 11) standalone platform highlighted 35 biosynthetic gene clusters (BGC). The production of valinomycin (NRP), alpiniamide (PK-NRP), and indigoidine (NRP) was confirmed by metabolite profile evaluation and heterologous host reconstitution (12, R. Sigrist, L. Gonzaga de Oliveira, 23 August 2017, Brazilian patent application BR1020170130239). This whole-genome sequencing reveals that the genome of endophyte Streptomyces sp. strain CBMAI 2042 encodes a large reservoir that qualifies for further biotechnological studies.

### Data availability.

This whole-genome shotgun project has been deposited at DDBJ/ENA/GenBank under the accession number RCOL00000000, BioProject number PRJNA487706, BioSample number SAMN09908177, and SRA number PRJNA487706 (SRA runs SRR8038340 to SRR8038346). The version described in this paper is the first version, RCOL01000000. Streptomyces sp. strain CBMAI 2042 was deposited at the Brazilian Collection of Environmental and Industrial Microorganisms (CBMAI).
